# Clinico-epidemiological profile of *Acinetobacter* and *Pseudomonas* infections, and their antibiotic-resistant pattern in a tertiary care center, Western Nepal

**DOI:** 10.3126/nje.v9i4.26962

**Published:** 2019-12-31

**Authors:** Shankar Baral, Anjila Pokharel, Supram Hosuru Subramanya, Niranjan Nayak

**Affiliations:** 1 Department of Internal Medicine, Manipal College of Medical Sciences, Pokhara, Nepal; 2 Department of Microbiology, Manipal College of Medical Sciences, Pokhara, Nepal

**Keywords:** *Acinetobacter spp.*, *Pseudomonas aeruginosa*, multi-drug resistance, antibiotic susceptibility

## Abstract

**Background:**

Infections caused by *Acinetobacter* species and *Pseudomonas* species, especially multidrug-resistant (MDR) strains pose a serious management challenge with a public health threat.

**Materials and Methods:**

A hospital-based retrospective study of patients who were infected with *Acinetobacter* spp or *Pseudomonas aeruginosa* was carried out at Manipal Teaching Hospital from 2014 to 2016.

**Results:**

A total of 170 cases of infections with *Acinetobacter* spp. and 313 cases with *Pseudomonas aeruginosa* were studied. The rate of nosocomial infections was higher than non-nosocomial infections. ICU was found as the major hub for both the organisms; (53.5% of cases due to *Acinetobacter* spp. and 39.6% due to *Pseudomonas aeruginosa*). Most isolates were of respiratory tract origin (*Acinetobacter* 74.7% and *Pseudomonas aeruginosa* 65.8%). Percentage resistance of *Acinetobacter* spp. towards polymyxin B was found to be quite low (18.8%). Similarly, resistance rates of *Pseudomonas aeruginosa* against amikacin were also found to be low, i.e., 17.4%. A higher prevalence of multidrug resistance was seen among *Acinetobacter* spp than among *Pseudomonas aeruginosa* (75.9% vs. 60.1%). The hospital stay was longer for patients infected with MDR isolate (p=0.001 for *Acinetobacter* spp. and p=0.003 for *Pseudomonas aeruginosa*). The mortality rate was higher in infections due to *Acinetobacter* spp (15.9%) as compared to *Pseudomonas aeruginosa* (8.3%).

**Conclusion:**

This study reveals that infections caused by *Acinetobacter* species and *Pseudomonas aeruginosa* are associated with prolonged hospital stay and high in-hospital mortality. These emphasize the need for prudent use of antibiotics and aggressive infection control strategies.

## Introduction

*Acinetobacter* and *Pseudomonas* species are gram-negative bacilli that commonly cause healthcare-associated infection. These can survive for prolonged periods in the environment and the hands of healthcare workers [1] and can cause nosocomial infections in critically ill patients with breaches in skin and airway integrity and on catheterization [2]. Infections due to multidrug-resistant (MDR) *Pseudomonas aeruginosa* and *Acinetobacter* spp. are believed to result in higher mortality, prolonged hospital stay, and higher health care costs as compared to those caused by antibiotic susceptible bacteria. Given the range and diversity of resistance patterns among clinical isolates of *Acinetobacter* spp. and *Pseudomonas aeruginosa*, therapy should be guided on the basis the results of adequately performed antimicrobial susceptibility testing [1, 2]. Antibiotic selection for empirical therapy is challenging and must rely on recent institutional-level susceptibility data. Unfortunately, at this stage, very little information is available on such therapeutic pipeline. Although drug resistance in *Acinetobacter* and *Pseudomonas* is a recognized problem in Asia, including Nepal [3,4], the effect of MDR *Acinetobacter* and *Pseudomonas* infections on the therapeutic outcome in patients is yet to be determined. We, therefore, undertook this study to evaluate the clinico-epidemiological profile of *Acinetobacter* spp. and *Pseudomonas aeruginosa* infections and to find out the current trend of drug resistance amongst these bacteria in a tertiary care center of the western region of Nepal. Additionally, we attempted to determine the outcome of infections caused by MDR *Acinetobacter* spp. and *Pseudomonas aeruginosa* on the mortality rates and length of hospital stay of patients that could have direct implications on the health care costs

## Methodology

### Study design and participants:

This was a hospital-based retrospective observational study conducted in Manipal Teaching Hospital, a 750 bedded tertiary healthcare center in the western region of Nepal. Specimens were obtained from the lower respiratory tract, blood, urine, pus, and other body fluids according to the guidelines recommended by the American Society of Microbiology [5]. Isolates of *Acinetobacter* spp. and *Pseudomonas aeruginosa* from the above mentioned clinical specimens of hospitalized patients over three years (from November 2014 to November 2017) were studied. Nosocomial isolates were defined as those grown from specimens that were sampled after 48 hours of hospitalization.

The case fatality rate was calculated by dividing the number of deaths from a specified disease over a defined period by the number of individuals diagnosed with the disease during that time; the resulting ratio is then multiplied by 100 to yield a percentage.

#### Laboratory identification:

The specimens had been cultured on Chocolate agar (CHA), 5% Sheep Blood agar (BA), and MacConkey agar (MA) plates. Organisms were identified, and their clinical significance was judged following standard microbiological techniques after interpreting microscopic findings, colony morphology, and biochemical properties [5].

#### Antibiotic susceptibility testing (AST):

Antimicrobial susceptibilities of all the isolates were determined by the Kirby-Bauer disc diffusion method, as recommended by the Clinical and Laboratory Standards Institute (CLSI 2014) [6]. *E. coli* ATCC 25922 and *Pseudomonas aeruginosa* ATCC 27853 were used as controls. Multidrug resistance was defined according to the current guidelines [7].

### Data collection

#### Patient data:

Medical and demographic data of hospitalized patients with culture-positive *Acinetobacter* spp. and *Pseudomonas aeruginosa* were retrieved from patient’s medical records. Data that were recorded include age, gender, ward location, duration of hospitalization, date of specimen collection, specimen site, type of specimen, and date of demise, if any. Microbiological data were obtained from the laboratory records.

#### Questionnaire:

No questionnaire was included in the study protocol.

#### Inclusion criteria:

Patients whose sputum, blood, urine, pus, and other body fluids yielded *Acinetobacter* spp. and *Pseudomonas aeruginosa* (non-repeating isolates) were included in the study.

#### Exclusion criteria:

Those patients whose specimens grew more than one isolate and whose records did not reveal complete data during the study were excluded.

#### Sample size calculation:

All 483 cases (yielding as many numbers of isolates) were investigated by the statistical parameters for the convenience of calculations.

#### Outcome variable:

Outcome variables included the rates of isolation of *Acinetobacter* spp. and *Pseudomonas aeruginosa* and prevalence of MDR strains among organisms causing either nosocomial or non-nosocomial infections.

#### Explanatory variable:

These included demographic factors such as age, gender, and source of isolation

### Ethical committee approval:

Permission to conduct the study was obtained from the Institutional ethics and research committee, Manipal College of Medical Sciences, Pokhara.

### Data management and statistical analysis:

Data were analyzed using Microsoft Office Excel 2007, SPSS 11.5.

## Results

### Isolation of the organisms from various sources

**Figure 1:**
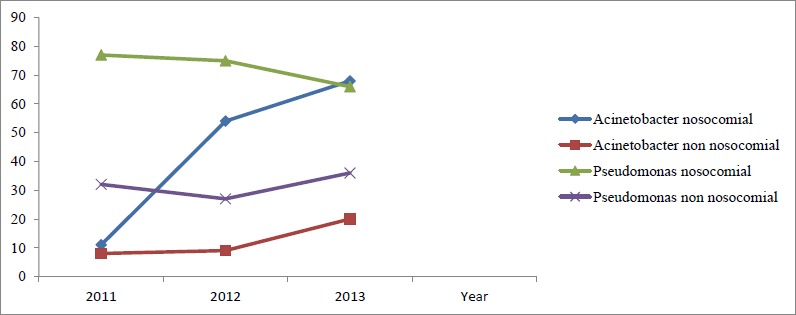
Yearly incidence of *Acinetobacter* and *Pseudomonas* species

A total of 483 cases were studied, of which specimens from 170 cases grew *Acinetobacter* spp. and those from the remaining 313 cases grew *Pseudomonas aeruginosa*. As depicted in figure 1, *Acinetobacter* infection was found to be on an increasing trend over the years. However, no such rising trend was noticed in *Pseudomonas aeruginosa* infection. Nosocomial infections due to both the organisms were found higher (Figure 1). Both isolates were detected with high frequency among the elderly males. The demographic characteristics of the study population are shown in table 1 and table 2. Most of the isolates were of respiratory tract origin (74% for *Acinetobacter* spp and 65.8% for *P. aeruginosa*; table 1.

### Antibiotic Resistant Pattern:

**Figure 2.**
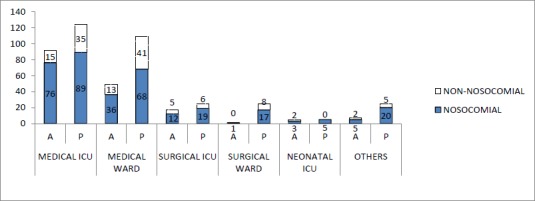
Distribution of *Acinetobacter* and *Pseudomonas* species in various departments

As shown in figure 2, the majority of the nosocomial isolates of both *Pseudomonas aeruginosa* and *Acinetobacter* spp. were obtained from patients in ICUs rather than from the general wards. Overall, the nosocomial isolates outnumbered the non-nosocomial (community) isolates in all categories of patients. *Acinetobacter* isolates among the ICU patients accounted for 53.5% [91(76 from medical ICU, 12 from surgical ICU, and 3 from neonatal ICU) of 170] and *Pseudomonas aeruginosa* for 36.1% [113(89 from medical ICU, 19 from surgical ICU, and 5 from neonatal ICU) of 313] of the cases (Figure 2).

Antibiotic susceptibility patterns of the organisms are depicted vide table 1. Percentage resistance of *Acinetobacter* isolates ranged between 63-97.6% against the majority of the antibiotics tested. However, cefoperazone-sulbactam, meropenem, and polymyxin B showed promising effects, resistance rates being 48.4%, 38.2%, and 18.8%, respectively. In the case of *Pseudomonas aeruginosa*, however, results were relatively encouraging, resistance rates varied between 17.4% to 33.4% against amikacin, ciprofloxacin, gentamicin, piperacillin-tazobactam, polymyxin B, meropenem and cefoperazone-sulbactam. Percentage resistance against other antibiotics, however, varied between 47.85 to as high as 87.4%.

It is noteworthy to mention that quite a high proportion (75.9%) of *Acinetobacter* species were MDR. Comparatively, MDR *Pseudomonas aeruginosa* isolates were less in number, amounting to only 60.1% (p value=0.79). Amongst all *Pseudomonas aeruginosa* isolates, however, a significant proportion, i.e., 285 (91.2%) were resistant to cefixime, whereas very few (54; 17.4%) showed resistance to amikacin. Antibiotic susceptibility pattern is shown in table 1.

### The outcome of infection:

The total length of hospital stay and duration of stay after the index day (the day when culture showed positivity) were longer among patients yielding MDR bacteria than those yielding non-MDR bacteria on culture. Mortality was higher among patients infected with *Acinetobacter* spp. than with *P. aeruginosa* (15.9% vs. 8.3 %) [p value=0.085 for *Acinetobacter* spp. & 0.064 for *P. aeruginosa*). The mortality rate was two-fold higher among those infected with MDR organisms (table 3) when compared to those with non-MDR organisms.

## Discussion

### Etiological Trends and Patterns of Antimicrobial Resistance

*Pseudomonas aeruginosa* ranked second among the gram-negative pathogens reported to the National Nosocomial Infections Surveillance System (NNIS) [8] in causing hospital-acquired infections. The rate of Pseudomonal infection was high but remained static throughout our study period. On the other hand, infections caused by *Acinetobacter* spp were on an increasing trend each year. *Acinetobacter* species caused 7% of ICU healthcare-associated pneumonia in 2003 compared with 4% in 1986, as reported by the National Nosocomial Infections Surveillance System [9]. A similar trend was also found in studies conducted in Nepal by Mishra et al. [10]. *Acinetobacter* spp. has been associated with infections in critical care patients, especially with ventilator-associated pneumonia. In complying with other studies, we also found a high rate (78.2%) of nosocomial infections was due to *Acinetobacter* spp. [11]. As reported earlier [3], most of the nosocomial infections among the critically ill patients in our hospital were detected in the ICUs, followed by the medical wards. According to our observations, a total of 75.9% (127 out of 170) of the patients were infected with MDR *Acinetobacter* spp. This is in contrast to the findings of Mishra et al. [10], who noted that 95.16% of their patients from a referral hospital in Nepal were infected with MDR *Acinetobacter* species. Such a high rate of detection of MDR isolates in their study was attributed to Extended-spectrum β-lactamase (ESBL) and AmpC beta-lactamase production [10]. The discrepancy in the isolation rates of MDR *Acinetobacter* spp. between our study and theirs’ could be due to the differences in adapting the antibiotic policies over the period.

Presently, fluoroquinolone resistance is being recognized as an emerging problem in *Acinetobacter* species worldwide. We found ciprofloxacin resistance in 67.6% of the isolates. This is in agreement with the results of Joshi et al. from India [12], and Mishra et al. from Nepal [10] who reported 72.9% and 64.52% fluoroquinolone resistance respectively among their *Acinetobacter* isolates. By the observations noted above [10, 13-16], it is obvious that the emergence of MDR *Acinetobacter* amongst the inpatients is always challenging before the clinician. In this context, other options, such as beta-lactam and beta-lactamase inhibitor combinations, could be the alternative therapeutic regime. We explored the scenario of the susceptibility pattern of the *Acinetobacter* spp. towards beta-lactam beta-lactamase inhibitor combinations. It was revealed that 48.4% of the *Acinetobacter* spp. were resistant to cefoperazone-sulbactam and 63% to piperacillin-tazobactam. Ling et al. [14] from Shanghai noted that 91% of *Acinetobacter* were susceptible to cefoperazone-sulbactam combination than to piperacillin-tazobactam (91% vs. 21%).

Resistance rate of *Acinetobacter* spp. towards carbapenem (38.2%) was comparable to that reported from India (34%), although a recent study from Nepal documented almost 50% of the clinical isolates to be resistant to this drug [10]. However, resistance rates towards 3rd generation cephalosporins, as shown by us (>80%) were in agreement with the observations of Mishra et al. [10]. Considering the above, a combination of meropenem or cefoperazone-sulbactam or piperacillin-tazobactam with amikacin could be the best option for cases infected with MDR infections in our setting.

It was encouraging to note that a maximum number (138, i.e., 81.2%) of *Acinetobacter* isolates were susceptible to Polymyxin B. Though this drug is often, not preferred in clinical practice due to its nephrotoxicity and neurotoxicity [15] yet there are scanty reports in favor of its efficacy against this organism [16]. Nevertheless, this needs further evaluation in clinical practice. Moreover, recent studies even documented less toxicity, possibly because of lower dosage, different drug formulations, and careful patient monitoring; nephrotoxicity rates accounting up to 36%, and neurotoxicity being far less common [15]. Tigecycline and colistin are drugs that have been active against most MDR strains of *A. baumannii* [17]. But both of these drugs are not under clinical use in our setting.

We found that patients having MDR *Acinetobacter* infections had increased length of hospital stay as compared to those infected with non-MDR *Acinetobacter* (19 days vs. 13 days). This finding combined with increased risk for in-hospital transmission of the organism [18] supports recommendations to implement aggressive control measures to limit the transmission of MDR *Acinetobacter* spp. in health care settings. In the present study, as high as 15.9% mortality rate was noted among the cases infected with MDR *Acinetobacter*. Sunenshine et al. [13] observed slightly higher mortality (26%) in their hospitalized patients who had infections due to MDR strains of *Acinetobacter*.

### Impact of drug resistance on hospital stay and patient management

Most of the *Pseudomonas aeruginosa* isolates were from the Medical ICU (39.6%). Our result was in consistence with the previous studies [8, 19]. Multidrug resistance is a known clinical problem with *Pseudomonas* spp. with a direct impact on mortality. Infections caused by *Pseudomonas aeruginosa* is frequently life-threatening and often difficult to treat because of the intrinsic susceptibility of *Pseudomonas aeruginosa* only to a limited number of antimicrobial agents [20, 21]. A study from Kathmandu, Nepal, showed 76.2% of *P. aeruginosa* isolates to be MDR [4]. We, however, noted that 60.1% of our *Pseudomonas aeruginosa* were MDR. This discrepancy could be because the organisms tested in the Kathmandu study [4] were from the ICU patients alone, whereas those studied by us included bacteria not only from the ICU patients but from other medical care units as well.

Whereas 82.6% and 80% of *Pseudomonas aeruginosa* were susceptible to amikacin and piperacillin-tazobactam respectively, only 8.8% had shown susceptibility towards cefixime. Bhandari et al. [22] from Nepal reported 84.8% of their *Pseudomonas aeruginosa* as susceptible to cefoperazone-sulbactam, 54.5% to piperacillin-tazobactam, and 51.5% to meropenem. They found a maximum number of the organisms showing resistance to cefixime (93.9%), a finding very much similar to ours (8.8% susceptible, 91.2% resistant). The length of hospital stay was higher for patients having infections due to MDR *Pseudomonas aeruginosa* than due to non-MDR *Pseudomonas aeruginosa* (21 days vs. 13 days). Previous workers also reported similar findings [23]. In our study, though the overall mortality was 8.3% that among the cases due to MDR strains was slightly higher (11.7%). The mortality among patients infected with MDR *Pseudomonas aeruginosa* was shown to be 21% in another study conducted by Gyanes et al. [24].

## Conclusion

This study reveals that infections caused by *Acinetobacter* species and *Pseudomonas aeruginosa* are associated with prolonged hospital stay and high in-hospital mortality. These emphasize the need for prudent use of antibiotics and aggressive infection control strategies.

### Strength of the study:

Our study provided adequate information on the high prevalence of MDR, as well as pan drug-resistant *Acinetobacter* and *Pseudomonas aeruginosa* infections among ICU patients in our setting.

### Limitation of the study:

1. Ample informative data on patients’ clinical details were not available to see any correlation between multidrug resistance and type of the organism, with the severity of illness
2. Susceptibility towards antibiotics like tigecycline and colistin were not tested.

### Future scope of the study:

Antibiotic resistance, including multidrug resistance, is the leading cause of mortality and morbidity in hospitalized patients. Further initiatives are needed to run continuous surveillance programs to monitor drug resistance patterns among these isolates to formulate measures for effective control of antibiotic resistance.

### What is already known on this topic:

Few studies on antibiotic resistance patterns of *Acinetobacter* and *Pseudomonas* are available from different parts of Nepal.

### What this study adds:

The study adds the information on the current antibiogram profile of *Acinetobacter* spp. and *Pseudomonas aeruginosa* isolated from hospitalized patients.

## Figures and Tables

**Table 1:** Characteristics of study population, specimen site and antibiotic resistant pattern

Variables	*Acinetobacter* spp. (n=170)**	*Pseudomonas aeruginosa* (n=313)**
Age	57.2 (21.86)	60.3 (20.43)
Male	93(54.7%)	191 (61%)
Female	77(45.3%)	122(39%)
Nosocomial	133(78.2%)	218(69.6%)
Non-nosocomial	37(21.8%)	95(30.4%)
**Distribution of isolates in relation to specimen sites**		
Respiratory tract	127(74.7%)	206(65.8%)
Blood	7(4.1%)	32(10.2%)
Soft tissue/wound	9(5.3%)	23(7.3%)
Urinary tract	20(11.8%)	21(6.7%)
Others	7(4.1%)	31(9.9%)
**Antibiotic resistant pattern**		
Cotrimoxazole	80.6%	82.3%
Ciprofloxacin	67.6%	22.6%
Gentamycin	74.1%	23.2%
Amikacin	65.3%	17.4%
Cefixime	97.6%	91.2%
Ceftriaxone	88.2%	65.0%
Cefotaxim	97.1%	70.8%
Ceftazidime	83.5%	58.4%
Cefipime	81.8%	47.8%
Cefoperazone-Sulbactum	48.4%	33.4%
Amoxicillin-clavulinic acid	95.9%	87.4%
Piperacillin-Tazobactum	63%	20.0%
Meropenem	38.2%	25.8%
Polymixin B	18.8%	25.1%

**Table 2:** Demographic characteristics of patients with multidrug-resistant (MDR) *Acinetobacter* and *Pseudomonas* infection vs. those with susceptible *Acinetobacter* and *Pseudomonas* infection

Characteristic	A^1^ n= 129 (75.9%)	A^2^ n=41 (24.1%)	P values for A^1^ vs A^2^ n= 170	Pa^1^ n=188 (60.1%)	Pa^2^ n=125 (39.9%)	P values for Pa^1^ vs Pa^2^ n=313
**Mean age, y**	57.93	56.89	0.77	59.11	60.43	0.79
**Age range, y**	1 day-84 years	1 day-91 years	--	15-81 years	1day-102 years	--
**Sex, male %**	60.3	51.8	0.288	66.7	60.7	0.61

**Legend: A^1^**- MDR Acinetobacter Spp.; **A^2^**- Susceptible *Acinetobacter* Spp.; **Pa^1^**- MDR *Pseudomonas aeruginosa*; **Pa^2^**- Susceptible *Pseudomonas aeruginosa.*

**Table 3:** Outcome of infection in patients infected with MDR vs susceptible organisms

Outcome evaluated	A^1^, n=114	A^2^, n=36	p values for A^1^ vs. A^2^, n=150	Pa^1^, n=179	Pa^2^, n=110	p values for Pa^1^ vs. Pa^2^, n=289
**Mean length of hospital stay (SD)**	19.36 (12.89)	12.89 (11.8)	0.001	21.24 (16.91)	13.3 (10.6)	0.003
**Mean length of hospital stay after index day (SD)**	15.43 (7.55)	7.85 (8.11)	<0.001	20.39 (14.44)	8.53 (8.53)	<0.001
**Mortality %, (n)**	18.6%, (24/129)	7.3%, (3/41)	0.085	11.7%, (21/179)	5.0%, (5/100)	0.064

**Legend: A^1^**- MDR Acinetobacter Spp.; **A^2^**- Susceptible Acinetobacter Spp.; **Pa^1^**- MDR*P aeruginosa*; **Pa^2^**-Susceptible *P aeruginosa.*
